# Relationships between colostrum supply of suckling piglets and *Salmonella* prevalence in piglet rearing

**DOI:** 10.1186/s40813-018-0085-6

**Published:** 2018-05-02

**Authors:** Anton Schulte zu Sundern, Carolin Holling, Karl Rohn, Josef Schulte-Wülwer, Ansgar Deermann, Christian Visscher

**Affiliations:** 1 Institute for Animal Nutrition, University of Veterinary Medicine Hannover, Foundation, Bischofsholer Damm 15, D-30173 Hannover, Germany; 2 Swine Health Service, Chamber of Agriculture Lower Saxony, Sedanstr. 4 D-26121, Oldenburg, Germany; 3 Institute for Biometry, University of Veterinary Medicine Hannover, Foundation, Bünteweg 2, D-30559 Hannover, Germany; 4 EVH Select GmbH, An der Feuerwache 14, D-49716 Meppen, Germany

**Keywords:** *Salmonella*, Health status, Fertility, Piglet rearing, Monitoring, Colostrum

## Abstract

**Background:**

Despite years of effort, the proportion of serologically *Salmonella*-conspicuous pig farms has not been significantly reduced. Incoming piglets are considered to be a significant source of *Salmonella* for feeder-to-finish-farms. Therefore it is important for farrow-to-feeder-farms to deliver *Salmonella*-inconspicuous piglets. The aim of the present study was to establish a possible link between an inadequate colostrum supply as a side effect of steadily increasing number of piglets born alive and weaned per sow and increasing *Salmonella* seroprevalence in piglet rearing on *Salmonella*-conspicuous farms.

**Methods:**

Twenty four farms in total were selected for this study. Half of the farms (*n* = 12) had been detected as *Salmonella*-conspicuous in previous serological tests on piglets (25 kg) and remaining farms (n = 12) had appeared *Salmonella*-inconspicuous. Every farm was visited once 24–28 h after the main day of farrowing. For sampling, four sows were randomly selected on each farm. The parity, the litter weight and the litter size were recorded. The sow and six of her piglets were selected for blood sampling (two light-weight, two medium-weight and two heavy-weight piglets respectively). In addition, the colostrum supply of newborn piglets was estimated by using the immunocrit.

**Results:**

The lightest piglets on *Salmonella*-inconspicuous and *Salmonella*-conspicuous farms showed a significant difference (*p* < 0.0339) in the colostrum supply (estimated by immunocrit). While light-weighted piglets in *Salmonella*-inconspicuous farms had an average immunocrit of 0.100 (±0.04) light-weighted piglets in *Salmonella*-conspicuous farms had an average immunocrit of 0.087 (±0.04). There was no significant difference (*p* > 0.05) in the factors body weight, litter weight, parity and litter size.

**Conclusion:**

The study provides preliminary evidence that when comparing *Salmonella*-inconspicuous farms with *Salmonella*-conspicuous ones, the colostrum supply may be a critical factor that needs to be considered. The fact that there is no difference in body weight between the two groups of farms suggests that there may be differences in farrowing management and especially colostrum management. Further studies are now required to investigate what causes the various colostrum supply on the respective farms and what long-term effects the individual colostrum supply might have on *Salmonella* prevalence at abattoir.

## Background

In 2016, human salmonellosis was the second most common foodborne zoonosis in the European Union as a whole as well as in Germany [1, 2]. Although the absolute number of human salmonellosis cases reported by the Robert Koch Institute (RKI) in Germany decreased from more than 70,000 patients in 2001 to 12,962 in 2016 [3], pork received attention as being the cause of human salmonellosis [1]. *Salmonella* Typhimurium (*S.* Typhimurium) pork-associated human salmonellosis accounted for the second largest percentage of all RKI-reported cases (36%) [3]. The *Salmonella* monitoring programme, which was started in 2003 and adapted in 2007 to the Pig-Salmonella Regulation, obliges all pig farmers to participate in the Quality and Safety GmbH (QS) system. Sampling in this programme is usually carried out in abattoirs and classifies fattening farms into risk classes [4]. This can lead to marketing disadvantages and price reductions. Despite intensive efforts, the percentage of *Salmonella*-conspicuous farms in Germany could not be significantly reduced [5]. The QS - statistics have shown an almost unchanged picture in the last ten years. The percentage of farms classified into category III (> 40% positive samples) decreased only slightly from 5.4% in 2006 to 3.4% in 2017. The percentage of category II farms (21–40% positive samples) even increased in the same period from 14.7% to 20.0% [6, 7]. Experience from various field studies in which also hygienic well-managed farms were included, suggests that improving hygiene as the sole means of reducing *Salmonella* is not the only priority [8]. Of greatest significance for the entry and distribution of *Salmonella* in pig herds are carrier pigs [9]. The association between *Salmonella* seroprevalence in sows and the direct detection of *S.* Typhimurium in rearing piglets is well known, as is the association between the direct detection of *S.* Typhimurium in rearing pigs and increased *Salmonella* seroprevalence in fattening pigs [10, 11]. These findings suggest that a reduction in *Salmonella* prevalence can only be successful if the piglet producers are involved. In recent years, they have been able to achieve an enormous increase in reproductive performance. For example, an analysis performed among northern German piglet producers showed an increase from 11.10 live born piglets per litter in the marketing year 2006/07 to 13.91 in the marketing year 2015/16 [12]. This also presents piglet producers with new challenges. Increasingly large litters with low average birth weights require intensive care and good management. Schulte zu Sundern et al. [13] were able to demonstrate in comparative analysis of results of a health screening and results of computer-supported sow planning that farms with an above-average fertility performance (live born or weaned piglets) often do not belong to the farms with the lowest *Salmonella* seroprevalence of ready-to-sell piglets. It was also shown that the average number of weaned piglets had a greater influence on the *Salmonella* seroprevalence than the average number of piglets born alive. This suggests that management from birth to weaning could be critical for *Salmonella* prevalence on the farm. The focus of many studies is the colostrum supply in the first days of life. Quesnel et al. [14] were able to prove that the litter size is not directly related to the amount of colostrum which is produced. For very large litters, there may be a gap between the amount of colostrum produced and the amount that would be necessary for a sufficient supply of all piglets. This condition is intensified by the fact that the amount of colostrum produced varies between 2.8 kg / d and 8.5 kg / d [15]. The aim of the present study was to establish a possible link between an inadequate colostrum supply as a side effect of steadily increasing reproductive performance and increasing *Salmonella* seroprevalence in piglet rearing on *Salmonella*-conspicuous farms.

## Methods

The study was carried out in cooperation with EVH-Select GmbH, an association of six northern German piglet producer communities in which more than 250 piglet producers are organised. The data from a health status monitoring programme organised by EVH-Select GmbH was used retrospectively for this field study. Under the organisation of EVH-Select GmbH, this monitoring has taken place every six months since 2014 on the farms and provides information about the health status of the piglets to the feeder-to-finish-farms. Participation is voluntary. For sampling, ten piglets weighing 25 kg are used for each screening. The animals used for the sampling are randomly selected within an age group. Obviously sick and nursed animals are not selected. *Salmonella* LPS antibodies were detected by Herdcheck® *Salmonella* ELISA (IDEXX Laboratories, Hoofddorp, the Netherlands). The samples were considered “positive” if the optical density (OD) was ≥10%. The direct test for *Salmonella* is not part of this health-status-monitoring. On the basis of the available health-status-monitoring results, farms (*n* = 12) were selected (Table 1) that had been experiencing an increased *Salmonella* seroprevalence of ready-to-sell piglets for a longer period of time and that had consulted veterinarians for advice. For every single *Salmonella*-conspicuous farm one farm was selected (n = 12) comparable in hygiene, management, performance, farm size and veterinary care but inconspicuous in *Salmonella* seroprevalence. The farms C and F were assessed as *Salmonella*-inconspicuous despite striking health-status-monitoring results. The relatively high average values could be explained by very high individual values in older health-status-monitoring results.

**Table 1:** Results of the voluntary health-status-monitoring from 2014 to 2017 on *Salmonella*-inconspicuous and *Salmonella*-conspicuous farms

*Salmonella*-inconspicuous farms					*Salmonella*-conspicuous farms				
Farm	Average *Salmonella* - OD	Number of tests	Proportion of postive piglets	Proportion of postive piglets [%]	Farm	Average *Salmonella* - OD	Number of tests	Proportion of postive piglets	Proportion of postive piglets [%]
A	1.4	4	0/40	0	M	16.26	5	19/50	38
B	2.83	4	2/40	5	N	19.71	4	14/40	35
C	6.14	4	7/40	18	O	18.41	5	22/50	44
D	0.32	1	0/10	0	P	9.62	1	2/10	20
E	0.94	4	1/40	3	Q	18.08	5	21/50	42
F	22.35	7	16/70	23	R	14.73	7	24/70	34
G	1.64	5	2/50	4	S	8.01	5	13/50	26
H	1.84	5	1/50	2	T	9.09	5	17/55	31
I	1.00	3	0/30	0	U	11.57	7	26/70	37
J	2.38	5	4/50	8	V	15.01	6	22/60	36
K	3.00	4	4/40	10	W	22.28	5	29/50	58
L	0.39	6	0/60	0	X	16.05	5	18/50	36

### Animals

All participating piglet producers (*n* = 24) were located in the federal state of Lower Saxony in the districts of Emsland, Grafschaft Bentheim and Osnabruck. Only a small proportion of farms (*n* = 2) were farrow-to-finish farms. The remaining farms were exclusively piglet producers. The average number of sows kept was 309 sows (*Salmonella*-inconspicuous farms: 280, *Salmonella*-conspicuous: 339, respectively). The average number of piglets born alive and weaned per litter (Ø 12 month before sampling) was 13.87 and 11.98. (*Salmonella*-inconspicuous farms: 13.99 and 11.99, *Salmonella*-conspicuous: 13.76 and 11.97, respectively). The majority of the farms used sows from breeding lines of DanAvl® (*n* = 10). The remaining farms used sows from the breeding lines of the Bundes Hybrid Zucht Programm, Ellringen, Germany (BHZP®, *n* = 7), Topig’s Norsvin®, Senden, Germany (*n* = 3) or Pig Improvement Company Deutschland GmbH, Hannover, Germany (PIC®, *n* = 4).The large proportion of farms produced at three-weekly intervals (*n* = 9), followed by those producing at fortnightly intervals (*n* = 6), at weekly intervals (n = 4) and others (*n* = 5). The average suckling time was 25.25 days (*Salmonella*-inconspicuous 24.91 days, *Salmonella*-conspicuous 25.58 days). The sows selected for the study had on average 5.03 parities (*Salmonella*-inconspicuous 4.78 ± 2.48, *Salmonella*-conspicuous 5.27 ± 2.14, respectively). The following boar lines were used, listed in decreasing order of importance PIC® 408 (*n* = 8), db.77® (n = 6), German Pietran® (*n* = 3), Topigs® (*n* = 3). Four farms used different boar lines.

### Sample collection

All farms (*n* = 24) were visited once depending on their production rhythm 24–48 h after the main farrowing day. On each farm, four sows were randomly selected from all sows already farrowed. A uniform selection of sows was not possible. Due to different herd sizes the total number of sows (24-48 h after farrowing) was totally different. But if possible one of the selected sows was first parity. Foster-mother sows and sows with unfamiliar piglets were not included in the selection. Recorded were the parity, the litter size and the total weight of the litter. For blood sampling, six piglets per litter were selected. The selection of the piglets was made in such a way that two light-weight, two medium-weight and two heavy-weight piglets were always used for sampling in relation to the litter. The individual weight of the selected piglets was recorded, too. On 19 farms blood sampling also included the respective maternal sows (*n* = 71); (12 *Salmonella*-inconspicuous farms, seven *Salmonella*-conspicuous farms). In order to ensure the comparability of the serological results despite different sample numbers, seven additional pairs were formed between the categories (seven *Salmonella*-inconspicuous farms, seven *Salmonella*-conspicuous farms). For the sample collection Serum Monovette with coagulation activator were used (Monovette 9 mL, Sarstedt AG & Co., Nümbrecht, Germany). The collected blood samples were refrigerated, transported to the laboratory and centrifuged at 2000 x g for 10 min, and the serum samples stored at − 20 °C until further analysis.

### Analysis

The samples were serologically examined using standardised methods in an accredited laboratory (Vaxxinova diagnostics GmbH, Leipzig, Germany). The detection of *Salmonella* LPS antibodies was carried out as in the health-status-monitoring using Herdcheck® *Salmonella* ELISA (IDEXX Laboratories, Hoofddorp, the Netherlands). The cut-off for the examined sows was carried out in accordance with the requirements of the Pig *Salmonella* Regulations for slaughter pigs. The samples of the examined sows were regarded as “serologically positive” if the optical density (OD) was ≥40%. The suckling piglets were not classified into “serologically positive” or “serologically negative” groups. The quantification of the colostrum supply of the piglets was carried out by means of the immunocrit method [16]. For this, 50 μL of serum were mixed with 50 μL of 40% (wt / vol) ammonium sulphate. The Ig present in the serum was precipitated. This was followed by centrifugation at 12000×g in a hematocrit capillary (disposable microhaematocrit capillary tubes 75 mm / 75 μL, Hirschmann Laborgeräte GmbH & Co. KG, Eberstadt, Germany) for 10 min. The resulting precipitate in relation to the total volume allows the colostrum supply to be estimated.

### Statistical analysis

The statistical analysis of the data was carried out with the statistical analysis program SAS®9.4 for Windows, using the SAS® Enterprise Guide®, Client Version 7.1 (SAS Institute Inc. Cary, USA). By means of the Shapiro-Wilks test, the quantitative parameters were checked for normal distribution. For the normally distributed parameters immunocrit and body weight, possible differences between inconspicuous and conspicuous farms for the three weight categories were tested by the t-test for independent samples. The comparison between inconspicuous and conspicuous farms for non-normally distributed *Salmonella* antibody results was performed using the Wilcoxon 2-Sample test. A significance level α of 5% (*p* < 0.05) was determined. For the correlation analysis of normally distributed data the correlation coefficient of Pearson was used. For non-normally distributed data sets, the Spearman rank correlation coefficient was calculated. Interpreting the correlation coefficient Rho was determined as follows: 0.0 ≤ *r* ≤ 0.2 = no to low correlation; 0.2 < *r* ≤ 0.5 = weak to moderate relationship; 0.5 < *r* ≤ 0.8 = clear relationship; 0.8 < *r* ≤ 1.0 = high to perfect correlation.

## Results

### Serology

In the serological examination and the detection of *Salmonella* antibodies the average OD in the examined sows showed a significant difference (*p* < 0.0451) between those of *Salmonella*-inconspicuous and *Salmonella*-conspicuous farms. The average OD of sows selected for sampling was 45.43% (± 26.89) for the 12 *Salmonella*-inconspicuous farms. In the seven farms that were previously classified as *Salmonella*-conspicuous by sampling the ready-to-sell piglets, the average OD of the tested sows was 32.88% (± 21.96). When considering only the results of the sows of the 14 farms (seven *Salmonella*-inconspicuous farms, seven *Salmonella*-conspicuous farms), the difference was even greater (*p* < 0.0153). The mean OD of the sows on the seven *Salmonella*-inconspicuous farms was 50.85% (± 29.34) and on the seven *Salmonella*-conspicuous farms 32.88% (± 21.96). On evaluating the study results of the 14 farms, no serologically positive sow was detected on five farms (Table 2). Although there was a significant difference in the *Salmonella* seroprevalence of the sows, the serological results of the piglets were similar on *Salmonella*-inconspicuous and *Salmonella*-conspicuous farms (Table 3).

**Table 2:** On 14 farms (seven Salmonella-inconspicuous and seven Salmonella-conspicuous farms) four sows were tested by Salmonella antibodies

Farm	“Positiv”– tested sows on *Salmonella*-inconspicuous farms	Farm	“Positiv”-tested sows on *Salmonella*-conspicuous farms
A	2	M	1
B	4	N	1
D	4	P	1
G	0	S	1
H	0	T	2
I	0	U	0
J	1	W	0

The samples were regarded as “serologically positive” if the optical density (OD) was ≥40%

**Table 3:** Body weight (BW), Immunocrit value and Salmonella-OD of the tested piglets 24–48 h post natum (p.n.) divided into light-, medium- and heavy-weight piglets, *Salmonella*-inconspicuous and *Salmonella*-conspicuous farms

		Body weight [kg]		immunocrit		*Salmonella* - OD	
		*Salmonella*-inconspicuous farms	*Salmonella*-conspicuous farms	*Salmonella*-inconspicuous farms	*Salmonella*-conspicuous farms	*Salmonella*-inconspicuous farms	*Salmonella*-conspicuous farms
BW category	n-animals/ BW category	88	96	88	96	88	96
Light-weight		1.05 (±0.25)	1.05 (±0.29)	0.100^a^ (±0.04)	0.087^b^ (±0.04)	35.85 (± 38.66)	36.18 (± 39.31)
Medium-weight		1.38 (±0.25)	1.36 (±0.27)	0.107 (±0.03)	0.098 (±0.03)	38.71 (± 40.12)	37.59 (± 37.51)
Heavy-weight		1.69(±0.27)	1.78(±0.31)	0.114 (±0.03)	0.111(±0.03)	43.65 (± 41.88)	41.77 (± 38.55)

^a .b^averages differ significantly within a row (*p* < 0.05)

### Colostrum supply

On both the *Salmonella*-inconspicuous and *Salmonella*-conspicuous farms, the two selected light-weight piglets per litter had a significantly lower colostrum supply (estimated by the immunocrit) than their medium-weight and heavy-weight littermates. It was also shown in this study that on *Salmonella*-conspicuous farms the colostrum supply of the light-weight piglets in the litter was significantly worse (*p* < 0.0339) than in the group of the light-weight piglets on *Salmonella*-inconspicuous farms. While light-weighted piglets in *Salmonella*-inconspicuous farms had an average immunocrit of 0.100 (±0.04) light-weight piglets in *Salmonella*-conspicuous farms had an average immunocrit of 0.087 (±0.04). There was no significant difference between *Salmonella*-inconspicuous and *Salmonella*-conspicuous farms, in the colostrum supply of medium-weight and heavy-weight piglets. The average weights of the light-weight, medium-weight and heavy-weight piglets did not differ in the two categories (Table 3). It was also shown that the colostrum supply on *Salmonella*-conspicuous farms was weak to moderate dependent (*r* = 0.220) from piglet weight. No correlation could be found on *Salmonella*-inconspicuous farms between bodyweight and colostrum intake (*r* = 0.097). Furthermore there were no significant differences between Salmonella-inconspicuous and Salmonella-conspicuous farms in recorded litter weight, parity and litter size (Table 4).

**Table 4:** Average litter size, litter weight, parity and Salmonella-OD of the tested sows

	*Salmonella*-inconspicuous farms	*Salmonella*-conspicuous farms
Litter size	13.65 (± 2.10)	13.57 (± 2.91)
Litter weight [kg]	18.94 (± 3.99)	18.93 (± 3.51)
Parity	4.78 (± 2.48)	5.27 (± 2.14)
*Salmonella* - OD – Sow	45.43 (± 26.89)	32.88 (± 21.96)

## Discussion

### Classifying the farms

Classifying the farms into *Salmonella*-inconspicuous and *Salmonella*-conspicuous was based on a retrospective evaluation of health-status-monitoring. This monitoring was not performed on sows but on piglets (25 kg) and included only the indirect detection of *Salmonella* antibodies and not direct cultural *Salmonella* detection. The already established health-status-monitoring is based on the desire of the feeder-to-finish-farms to obtain information on the *Salmonella* status of the farrow-to-feeder farms. Comparing the inconspicuous (*n* = 12) and the conspicuous (n = 12) farms, it was found that the percentage of serologically positive sows was higher on those farms classified as inconspicuous (40.9%) than on those classified as conspicuous (29.6%). Furthermore, the average OD of the examined sows was higher on those farms classified as inconspicuous (OD 40.43%) than on those farms classified as conspicuous (OD 32.88%). These results raise the question whether the previous monitoring results, which focused on the sampling of piglets, provide a realistic picture of *Salmonella* prevalence for the entire herd (and the classification) into inconspicuous and conspicuous farms. In a pan-European study on *Salmonella* prevalence, Bole-Hribovšek et al. [17] found *Salmonella* on 31.8% of all studied farrow-to-feeder farms by direct detection. Meyer et al. [18] achieved similar results. In their study, carried out among northern German piglet producers of various forms of husbandry, they found at least one positive seroreactors among the sows examined in 71.8% of all conventional piglet producers studied. Overall, 12.3% of all sows tested were seropositive. The detection of *Salmonella* positive seroreactors on those farms classified as *Salmonella* inconspicuous farms is therefore not surprising. The spread of *Salmonella* in pig herds can be considered ubiquitous.

### Selection of animals

The animals selected for sampling were, two light-weight, two medium-weight and two heavy-weight piglets. The selection referred to the respective litter. A small percentage of individual animals were selected with a body weight of less than 1 kg. Some of these underweight animals, which had received only an insufficient amount of colostrum, were not successfully weaned and thereby played no role in the *Salmonella* distribution in the flat deck. Ferrari et al. [19] investigated the influence of birth weight and colostrum uptake (in g) on suckling pig mortality. While piglets with a birth weight of 1.40–1.45 kg and a colostrum intake of 250–300 g had a suckling pig mortality of 6.0% and 4.7% respectively, the mortality rate in 1.10–1.15 kg piglets and a colostrum intake of ≤150 g had a suckling pig mortality of 12.2% and 23.1%, respectively. High-performance farms are also able to raise the proportionately larger numbers of pigs, which are less developed at birth, through intensive management [20]. Despite losses among light or underserved piglets, many of these piglets are successfully weaned and could play a role in the infection in the flat deck. This is also supported by the findings of Schulte zu Sundern et al. [13] in a retrospective analysis of health status monitoring results and a comparison with data from computer-supported sow planning. They were able to prove that the most productive piglet producers were not among those with the lowest *Salmonella*-seroprevalence.

### Possible causes of a different colostrum supply

Both on the *Salmonella*-inconspicuous and *Salmonella*-conspicuous farms, the medium-weight and heavy-weight piglets in a litter were better supplied with colostrum than their light-weight littermates. The impact of birth weight on colostrum intake and the critical role played by light-weight piglets compared to their heavier littermates have been demonstrated in numerous studies ([16, 19, 21]). There was a significant difference (*p* = 0.033) in the colostrum supply of the lightest piglets between the *Salmonella*-inconspicuous and *Salmonella*-conspicuous farms. While the light-weight piglets on *Salmonella*-inconspicuous farms had an average immunocrit of 0.100 (± 0.04), the lightest piglets on *Salmonella*-conspicuous farms had only an average immunocrit of 0.087 (± 0.04). In the medium-weight and heavy-weight piglets, the difference between the inconspicuous and conspicuous farms was not significant (*p* = 0.199 and *p* = 0.591, respectively). In the data on litter weight and litter size, which also influence the colostrum supply as does birth weight ([15, 16]), no significant differences were found between the *Salmonella*-inconspicuous and *Salmonella*-conspicuous farms (Table 4). As the aforementioned biological factors do not cause the differing amounts in the colostrum supply it would appear that the farrowing-management or unrecorded factors play a decisive role therein. This is supported by the fact that, in our comparative analysis, the influence of weight on the colostrum supply on *Salmonella*-inconspicuous farms did not seem to be decisive (*r* = 0.097) whereas this factor was at least mild to moderate on *Salmonella*-conspicuous farms (*r* = 0.220). Factors that may explain the differences in colostrum supply of the light-weight piglets on *Salmonella*-inconspicuous and *Salmonella*-conspicuous farms are numerous. Declerck et al. [22] were able to show that the use of Oxytocin at birth and a long interval between births correlated negatively with the colostrum supply. Farmer and Quesnel [23] found numerous other factors in their review. In particular, the impact of on-demand sow feeding in the near-term and stress had a negative impact on colostrum formation. Finding the weak point in management for individual farms would be subject for further studies.

### Maternal-transmitting antibodies as effective protection against *Salmonella*

In our experiment, we found that every farm, both *Salmonella*- inconspicuous and *Salmonella*-conspicuous farms, a large percentage of the sows had *Salmonella* antibodies. This indicates a common spread of *Salmonella* on the farms. Effective protection of the piglets from *Salmonella* infection by vaccination of the sows was the aim of numerous experiments. The effectiveness thereof could be proved, for example [24]. In this previous study piglets from five sows were orally infected with a field strain on the fourth day of life and euthanised three days later. Two of the accompanying sows were vaccinated with an inactivated strain. Two more sows were classified as *Salmonella* negative by ELISA. The fifth selected sow had a high *Salmonella* antibody titer despite no vaccination. After piglet euthanasia, cultural studies on *Salmonella* were carried out. The piglets of the sows, which had either been vaccinated or, had high *Salmonella* antibody titers, showed a significantly lower number of *Salmonella* in the tested tissue. These findings are supported by the investigations by Roesler et al. [25]. Here, the use of an inactivated *Salmonella* vaccine in 25 sows also showed an effective reduction in *Salmonella* prevalence in piglet rearing. A similar result was found by Hur and Lee [26]. When considering the *Salmonella* antibodies detected by ELISA, it can be stated that despite differing amount of colostrum supply (measured by immunocrit), no significant differences in the average OD between the *Salmonella*-inconspicuous and *Salmonella*-conspicuous farms could be recognized. The complexity of the protection given by the colostrum intake does not appear to be fully ensured by sole consideration of the ELISA results. In addition to the immunoglobulins transmitted by the colostrum, other substances also appear to provide protection against *Salmonella* infection. Blais et al. [27] demonstrated a positive effect of colostrum-containing whey in their in vitro experiments. Their experiments utilised a porcine-intestinal-epithelial-cell (IPEC-J2) model, bovine colostrum and heat-killed (HK) *Salmonella* Typhimurium. The colostrum in the model was able to reduce the inflammatory processes caused by *Salmonella*, making it difficult to attach to the intestinal cells.

## Conclusion

The results of this field study suggest that in comparative investigations of *Salmonella*-inconspicuous and *Salmonella*-conspicuous piglet producers, inadequate colostrum supply of light-weight piglets could be a factor in increased *Salmonella* seroprevalence of piglets (25 kg) on *Salmonella*-conspicuous farms. Furthermore, no differences in birth weight, litter size, litter weight and parity between the *Salmonella*-inconspicuous and *Salmonella*-conspicuous farms could be determined. This suggests that there must be differences in management, especially between birth, weaning and sale. Based on this presumption, it must be examined in subsequent follow-up studies whether piglets with insufficient colostrum supply, at the end of rearing or slaughtering, appear conspicuous in their *Salmonella*-prevalence or whether piglets with sufficient colostrum supply appear inconspicuous at the same time in their *Salmonella* prevalence.

## Abbreviations

BW: Bodyweight.
HK: Heat-killed.
Ig: Immunoglobulin.
IPEC-J2: Porcine-intestinal-epithelial-cell.
LPS: Lipopolysaccharide.
OD: Optical density.
RKI: Robert Koch Institute

## References

[1] Anonym. EFSA scientific committee-scientific opinion on a quantitative microbiological risk assessment of salmonella in slaughter and breeder pigs. EFSA J. 2010:1547.

[2] Pfennigwerth N (2017). Bericht des Nationalen Referenzzentrums (NRZ) für gramnegative Krankenhauserreger.

[3] Anonym (2016). Berichte zur Lebensmittelsicherheit 2016: Zoonosen-Monitoring. Bundesamt für Verbraucherschutz und Lebensmittelsicherheit.

[4] Anonym: Verordnung zur Vermeidung der Salmonellenverbreitung durch Schlachtschweine (Schweine-Salmonellen-Verordnung vom 13. März 2007 (BGBl. I S. 322)), die zuletzt durch Artikel 137 des Gesetzes vom 29. März 2017 (BGBl. I S. 626) geändert worden ist. 2007.

[5] Rostalski A. Salmonella in pig farms. Limitations of counselling and alternatives to the exclusive control of slaughter pigs *Tierärztl Prax*. 2015;43:305–11. 10.15653/TPG-150734 26395467

[6] Römer R. Salmonellenmonitoringprogramm für die Fleischerzeugung- Aktuelle Trends und Herausforderungen für die Zukunft. In bpt Kongress Hannover 2016 Hannover bpt Akademie GmbH. 2016:92–6.

[7] May T: Salmonellenmonitoring. QS Qualität und Sicherheit GmbH; 2017.

[8] Roesner P, Eisenberg T, Hornstein O, Gebele U, Schulte-Wuelwer J, Schulze-Horsel T (2014). Salmonellen beim Schwein-Beratungsempfehlungen der Schweinegesundheitsdienste.

[9] Ahrens A: Epdemiologische Untersuchungen zum Vorkommen von Salmonellen bei sächsischen Mastschweinen mittels Fleischsaft-ELISA - Technik und bakteriologischer Untersuchungsmethodik nach der Amtlichen Sammlung von Untersuchungsverfahren nach § 35 LMBG. Universität Leipzig, Institut für Lebensmittelhygiene der Veterinärmedizinischen Fakultät; 2003.

[10] Kranker S (2001). Bacteriological and serological examination and risk factor analysis of salmonella occurence in sow herds, including risk factors for high salmonella seroprevalence in receiver finishing herds. Berl Munch Tierarztl Wochenschr.

[11] Hill AA, Simons RR, Kelly L, Snary EL (2016). A farm transmission model for salmonella in pigs, applicable to EU member states. Risk Anal.

[12] Anonym: Emslandauswertung 2016 - Ergebnisse und Auswertungen der Sauenplanerauswertung und Betriebszweigauswertung - Beratungsringe aus den Regionen Emsland, Grafschaft Bentheim und Ostfriesland; 2016.

[13] Schulte zu Sundern A, Rohn K, Holling C, Deermann A, Schulte-Wuelwer J, Visscher C (2017). Influence of increased fertility on the salmonella prevalence in piglets in pig-holding farms. Praktischer Tierarzt.

[14] Quesnel H, Farmer C, Devillers N (2012). Colostrum intake: influence on piglet performance and factors of variation. Livest Sci.

[15] Vadmand C, Krogh U, Hansen C, Theil P (2015). Impact of sow and litter characteristics on colostrum yield, time for onset of lactation, and milk yield of sows. J Anim Sci.

[16] Vallet J, Miles J, Rempel L (2013). A simple novel measure of passive transfer of maternal immunoglobulin is predictive of preweaning mortality in piglets. Vet J.

[17] Bole-Hribovšek V, Chriél M, Davies R, Fanning J, van de Giessen AW, Palancar LP, Ricci A, Rose N, Snow L: Analysis of the baseline survey on the prevalence of salmonella in holdings with breeding pigs in the EU, 2008: part a: salmonella prevalence estimates**.** European Food Safety Authority; 2008.

[18] Meyer C, große Beilage E, Krieter J (2005). Untersuchungen zur Salmonella-Seroprävalenz in unterschiedlichen Produktionssystemen beim Schwein. Tierarztl Prax Ausg G.

[19] Ferrari C, Sbardella P, Bernardi M, Coutinho M, Vaz I, Wentz I, Bortolozzo F (2014). Effect of birth weight and colostrum intake on mortality and performance of piglets after cross-fostering in sows of different parities. Preventive veterinary medicine.

[20] Boulot S, Quesnel H, Quiniou N (2008). Management of high prolificacy in French herds: can we alleviate side effects on piglet survival?. Proceedings of the 2008 Banff pork seminar; University of Alberta.

[21] Quesnel H (2011). Colostrum production by sows: variability of colostrum yield and immunoglobulin G concentrations. Animal.

[22] Declerck I, Sarrazin S, Dewulf J, Maes D (2017). Sow and piglet factors determining variation of colostrum intake between and within litters. Animal.

[23] Farmer C, Quesnel H (2009). Nutritional, hormonal, and environmental effects on colostrum in sows. J Anim Sci.

[24] Matiasovic J, Kudlackova H, Babickova K, Stepanova H, Volf J, Rychlik I, Babak V, Faldyna M (2013). Impact of maternally-derived antibodies against salmonella enterica serovar typhimurium on the bacterial load in suckling piglets. Vet J.

[25] Roesler U, Heller P, Waldmann KH, Truyen U, Hensel A (2006). Immunization of sows in an integrated pig-breeding herd using a homologous inactivated salmonella vaccine decreases the prevalence of salmonella typhimurium infection in the offspring. J Veterinary Med Ser B.

[26] Hur J, Lee J (2010). Immunization of pregnant sows with a novel virulence gene deleted live salmonella vaccine and protection of their suckling piglets against salmonellosis. Vet Microbiol.

[27] Blais M, Fortier M, Pouliot Y, Gauthier S, Boutin Y, Asselin C, Lessard M (2015). Colostrum whey down-regulates the expression of early and late inflammatory response genes induced by Escherichia coli and salmonella enterica typhimurium components in intestinal epithelial cells. Br J Nutr.

